# Maize *ZmHSP90* plays a role in acclimation to salt stress

**DOI:** 10.7717/peerj.15819

**Published:** 2023-10-03

**Authors:** Xinyan Yu, Yanxin Zhao, Yun Wang, Zhiqing Zou, Fenghai Li

**Affiliations:** 1 Shenyang Agricultural University, Shenyang, China; 2 Win-all High-Tech Seed Company Limited, Hefei, China; 3 Maize Research Center of Beijing Academy of Agriculture and Forestry Sciences (BAAFS), Beijing, China

**Keywords:** Salt stress, Transcriptome, Maize, Overexpression, Tobacco

## Abstract

**Background:**

Maize is sensitive to salt stress, especially during the germination and seedling stages.

**Methods:**

We conducted germination experiments on 60 maize materials under salt stress, and screened out the most salt-tolerant and salt-sensitive varieties based on germination indicators. Afterwards, transcriptome analysis was performed to screen for key regulators in the plumule and flag leaf at the germination and seedling stages, respectively. Following that, transgenic tobacco was developed to expose the roles and mechanisms of the candidate genes, enabling a deeper investigation of their functions.

**Results:**

Out of the 60 inbred lines of maize, “975-12” exhibits the highest level of salt tolerance, while “GEMS64” displays the lowest. The application of salt stress resulted in a significant increase in the levels of antioxidant enzymes in both “975-12” and “GEMS64”. ABA signal transduction and jasmonic acid pathways were the pathways that mainly affected by salt stress. In addition, a significant finding has been made indicating that when exposed to high levels of salt stress, the expression of *ZmHSP90* in ‘975-12’ increased while in ‘GEMS64’ decreased. Furthermore, in tobacco plants overexpressing *ZmHSP90*, there was an increase in antioxidant enzyme activity associated with salt tolerance. *ZmHSP90* enhanced the expression levels of *NtSOS1*, *NtHKT1*, and *NtNHX1* as compared to those in the salt treatment, causing the maintenance of Na^+^ and K^+^ homeostasis, suggesting that high expression of *ZmHSP90* was conducive to regulate Na^+^ transporters to maintain K^+^/Na^+^ balanced in tobacco.

## Introduction

Maize (Zea mays L.) is regarded as one of the three major cereal crops worldwide. Maize has strong adaptability and can be widely planted in fields in a wide range of global climate, soil and environmental conditions. Maize is sensitive to water, salt, and drought stress, all of which have become significant limiting factors for its development and yield (Cao et al., 2020a; Farooq et al., 2015). Salt stress can cause osmotic and ionic stress upon plants, and when stress is severe enough, it can cause the extravasation of plant tissue, whereby resulting in physiological dryness. Understanding the physiological and biochemical mechanisms in maize adapted to salt stress is critical for breeding salt-tolerant varieties (Zhang et al., 2020). Many genes have been found, cloned, and used as candidate genes in genetic engineering after being stimulated by salt stress. These proteins include late embryogenesis abundant proteins and key enzymes for osmolyte biosynthesis, as well as regulatory proteins involved in signal transduction regulation of stress-responsive gene expressions, such as transcription factors and protein kinases (Ali et al., 2021; Borkiewicz et al., 2020; Farooq et al., 2015; Wu et al., 2019).

Plant hormone signal transduction genes have been studied transgenically to improve plant stress tolerance. For example, transgenic Arabidopsis overexpressing *GsMYB15* improves the resistance to salt stress (Shen et al., 2018); what is more, the overexpression of *JAZ1* or *JAZ4* repressed the freezing stress response (Devireddy et al., 2021). Another study on Arabidopsis found that overexpressing *ERF74*, a member of the ethylene response factor VII protein family, enhanced drought tolerance by maintaining H_2_O_2_ homeostasis (Yao et al., 2017). Additionally, *PYR/PYL* overexpression in soybean conferred a higher tolerance to drought and salt stressors and enhanced the expression of the stress-responsive gene (Yu et al., 2021). The overexpression of some other genes also demonstrated strong stress resistance. The overexpression of HSP (Wang et al., 2016), IPT8 (Wang et al., 2015) and ZEP (Park et al., 2008) could effectively improve the stress resistance of plants. Therefore, understanding the molecular mechanisms of salt tolerance and screening maize salt tolerance genes are crucial for cultivating salt-tolerant maize.

Germination and early seedling growth were more sensitive to salinity than were later developmental stages (Farooq et al., 2015). Thus, salt tolerance studies during the germination and seedling stages could highlight the salt response process of maize. We analyzed the germination of 60 maize inbred lines and screened out suitable research materials for this study. After that, the mechanism through which genes respond to salt stresses in maize during the germination and seedling stage was examined. To investigate the functions of candidate genes further, we created overexpressed tobacco and performed salt stress tests to reveal the functions and mechanisms of the candidate genes.

## Methods

### Experimental design

**Figure 1:**
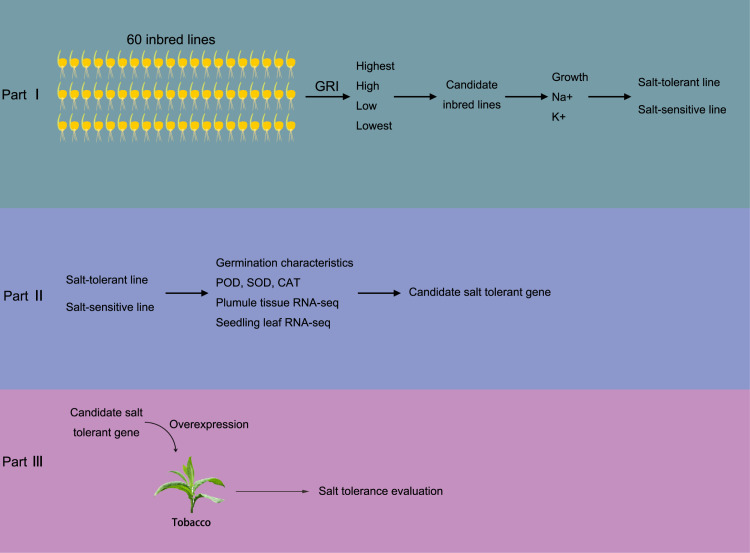
Brief sketch of the experimental design. The experiment is divided into three parts. In part I, 60 maize inbred lines were collected to test the Determination of Germination Index (GRI, GRI = germination rate of salt-treated seeds/germination rate of seeds under control condition). Plant materials with the highest, high, low, and the lowest GRI value were selected to preliminarily evaluate the salt tolerance. Then, salt resistance was further evaluated according the growth status, Na^+^ and K^+^ concentrations, according which the salt salt-tolerant and salt-sensitive maize materials were determined. In part II, the SOD, POD and CAT activity in both salt-tolerant and salt-sensitive maize were determined in germination and seedling periods. Also, transcriptome sequencing was carried out to screen salt-tolerant genes; In part III, the salt tolerance gene was transferred into tobacco and overexpressed to verify the salt tolerance of overexpressed tobacco.

At Shenyang Agricultural University’s maize breeding center, a total of 60 *Zea mays* L. plants were assessed for their resistance to salinity. The Seed Germination Rate-based salinity tolerance Index (GRI) was employed to evaluate the plants’ salinity tolerance, with the formula: GRI = germination rate of salt-treated seeds/germination rate of seeds under normal conditions. The germination process involved the use of different concentrations of saline culture medium for cultivation, with a control group (CK) established for comparison. The salt stress experiment utilized the same concentration gradient of salt stress as that used in Cao et al. (2020b). Next, maize lines with different salt tolerance grades were selected based on their GRI values. The growth status, as well as the concentrations of Na^+^ and K^+^, were measured as evaluation indexes for salt tolerance. This helped in determining the salt-tolerant and salt-sensitive maize materials. Subsequently, salt stress experiments were conducted on the selected materials. Maize plumule and seedling leaf tissues (third leaf stage) were collected for enzyme activity determination and transcriptome analysis. Based on the transcriptome data, candidate salt-tolerant genes were identified. To verify the function of these candidate genes, transgenic technology was employed using tobacco as the model organism. Figure 1 provides a schematic representation of the experimental design.

### Part I Salt tolerance evaluation

#### Salt treatment and sampling during the germination stage

Multiple germination tests were conducted in this study. Initially, the seeds of 60 maize varieties were subjected to a preliminary germination test under 100 mmol/L salt stress (three repetitions, with 150 seeds evaluated in each repetition). Subsequently, salt treatment experiments were conducted using different salt concentration gradients (0 mmol/L, 50 mmol/L, 100 mmol/L, and 150 mmol/L) to observe the impact of salt stress on germination characteristics of candidate salt-tolerant and salt-sensitive maize.

The germination experiment was performed with three replicates, each containing 30 seeds for each treatment. The seeds were placed on six layers of filter paper in a germinating disc and then transferred to a germination chamber with a temperature of 20 °C, 60% humidity, and a light/dark cycle of 8 h/16 h. The number of germinated seeds was counted on the 4th day for germination potential and on the 7th day for germination rate. Maize plumules were collected as plant samples, and the fresh samples were quickly frozen in liquid nitrogen and stored at −80 °C until salt resistance characteristics testing use.

### Salt treatment and sampling during the seedling stage

Maize seedlings at the three-leaf stage and with uniform growth and size were subjected to different salt concentration gradients (0 mmol/L, 50 mmol/L, 100 mmol/L, and 150 mmol/L) under normal growth conditions, including temperature, light, and humidity.

Plant samples were collected at the seedling stage (three-leaf stage) from the flag-leaf under different salt treatment conditions after 24 h and 48 h of salt stress. The fresh samples were immediately frozen in liquid nitrogen and stored at −80 °C until salt resistance characteristics testing use.

### Antioxidant enzyme activity determination

Firstly, leaf samples were collected and washed with a phosphate buffer solution (PBS) with a pH of 7.8. Then, the samples were homogenized using PBS with 4% polyvinylpyrrolidone (PVP) and 1 mM ethylene diamine tetraacetic acid (EDTA). The activities of SOD, CAT, and POD were determined using commercial assay kits. The T-SOD assay kit (hydroxylamine method, Cat. SCSP-503; Nanjing Jiancheng Corp., Nanjing, China), the Catalase (CAT) assay kit (Visible light, Cat. A007-1-1; Nanjing Jiancheng Corp., Nanjing, China), and the Peroxidase assay kit (colorimetry method, Cat. A084-3-1; Nanjing Jiancheng Corp., Nanjing, China) were used to determine the activities of SOD, CAT, and POD, respectively. Briefly, plant tissues were first homogenized using a plant tissue homogenizer (Laibei, Tianjin, China) and a refrigerated high-speed centrifuge (Eppendorf, Hamburg, Germany). After that, the SOD, CAT, and POD were measured using commercially available kits according to the manufacturer’s instructions. The activities of SOD, CAT, and POD were then calculated by dividing the total activity by the fresh weight. The experiment was conducted with three biological and three technical replicates.

### Na^+^ and K^+^ ions determination

The method used for determining the concentration of Na+ and K+ in the leaf samples was described by Zhao et al. (2014). Firstly, clean leaf samples were dried at 60 °C for 48 h and then ground into a fine powder in a dry mortar. The powder was then subjected to 12-hour nitric acid digestion, and the resulting solution was centrifuged. The supernatant was diluted to 5 times its volume, and the concentration of Na+ and K+ in the acid-digested tissues was measured using a flame photometer (Model 410 Sherwood, UK).

### Part II Transcriptome analysis

#### Tissue samples collection

To further investigate the response of salt-tolerant and salt-sensitive varieties to salt stress, transcriptome analysis was conducted at the germination and seedling stages.

During the germination stage, salt-tolerant and salt-sensitive maize were subjected to stress experiments at 0 mmol/L (marked as CK) and 100 mmol/L (marked as T) salt concentrations and plumule tissues were collected and stored at −80 °C until use.

During the seedling stage (at the three-leaf stage), the seedlings were randomly divided into three groups, each containing 5 seedlings. The salt treatment group for 24 h (recorded as 24h) and 48 h (recorded as 48h) were exposed to 100 mmol/L salt for 24 and 48 h, respectively. The CK group did not undergo salt stress treatment throughout the entire process. Flag leaf samples were collected at the 48th hour to minimize the interference of seedling development on transcriptome data. An implementation diagram of salt treatment during the seedling stage is presented in Fig. S1.

### RNA isolation and RNA-seq libraries preparation

Total RNA was extracted from frozen tissues using TRIZOL reagent (Invitrogen, USA), then purified and concentrated using the RNeasy MinElute Cleanup kit (Qiagen, Germany). The RNA-seq libraries were constructed with an Illumina TruSeq RNA sample preparation kit and TruSeq index adaptors. Sequencing was done on an Illumina Hiseq 4000.

### Transcriptome sequencing data analysis

The statistical power analysis for the given sample sizes was evaluated using the R package RNASeqPower version 1.18.0 (Hart et al., 2013). The transcriptome sequencing reads were mapped to the maize genome (https://www.maizegdb.org/genome/assembly/B73%20RefGen_v3) using TopHat2 (Kim et al., 2013), and transcripts were assembled and quantified using the Cufflinks pipeline (Trapnell et al., 2010). Differential gene expression analysis was performed using DESeq2 software with pairwise comparisons of identified genes with significant differences in expression between groups. Gene expression levels were quantified using reads per kilobase per million mapped reads (RPKM) values. Differentially expressed genes (DEGs) were screened based on the criteria of —log2(foldchange)—>1.5 and false discovery rate (FDR) adjusted *p*-value < 0.05. Up-regulated and down-regulated genes were defined as genes with higher and lower expression levels, respectively, in one group compared to another. Functional analysis was performed using the GO database (http://geneontology.org) and the KEGG database (http://www.genome.jp/kegg/), and Go terms and KEGG pathways were considered significantly enriched at an FDR < 0.05.

### Part III Salt tolerance gene transgenic verification

#### Isolation of candidate salt tolerance gene

Maize leaves were used to extract total RNA using a mini-BEST Universal RNA Extraction Kit (TAKARA, Shiga, Japan). The reverse-transcription PCR (RT-PCR) was performed with a PrimeScript™ RT reagent Kit 3.0 (TAKARA, Shiga, Japan). General PCR conditions were used to amplify the candidate salt tolerance gene. The PCR fragments were cloned into the pEASY-Blunt Simple Cloning Vector (TransGen Biotech, Beijing, China) and cultured on LB plates. The reconstructed plasmid was verified by Sanger sequencing.

### Subcellular localization

“The candidate salt tolerance gene was analyzed for subcellular localization using a vector encoding a GFP-tagged fusion protein as markers. To create the fusion proteins, the salt tolerance gene was cloned into the 16318 h GFP vector with a CaMV 35S promoter: green fluorescent protein (35S: GFP) cassette. The recombinant vector was validated by Sanger sequencing and then transformed into protoplasts using the previously described polyethylene glycol (PEG)-mediated transformation approach (Bart et al., 2006). The fluorescence was observed under an Olympus FluoView FV1000 confocal laser scanning microscope (Olympus, Japan) 16 h after transformation.

### Plasmid construction and *Agrobacterium tumefaciens* (*A. tumefaciens*)-mediated transformation of tobacco plants

The expression vector pCAMBIA3301, under the control of the CaMV 35S promoter (http://www.cambia.org), was used to clone the full-length cDNAs of the candidate salt tolerance gene. The recombinant vectors and a control pCAMBIA3301 were introduced into A. tumefaciens strains using the freeze-thaw method. The leaf disk transformation was performed based on a previous report (Curtis, Davey & Power, 1995). Briefly, the A. tumefaciens strain carrying the relevant plasmids was transformed into tobacco epidermal cells using the hand-infiltrating method. Three weeks later, the regenerated tobacco tissues were planted into tissue culture vessels containing medium. Positive transgenic plants were screened using qRT-PCR.

### Evaluation of salt tolerance in transgenic tobacco

Transgenic and wild-type (WT) tobacco plants were subjected to salt stress treatments. Seeds were initially planted in MS medium for growth assessment, and after three weeks, plants of the same size were transferred to MS medium containing 0, 100, 150, and 200 mM NaCl for 7 days. The plant biomass was estimated as fresh weight, and the activities of SOD, CAT, and POD were evaluated. In addition, the ROS content, Na^+^, and K^+^ ion concentrations in the leaves were also measured. We conducted qRT-PCR analysis on the expression levels of key regulatory genes, namely *NtSOS1*, *NtHKT1*, and *NtNHX1* investigate whether overexpression of *ZmHSP90* has any impact on SOS pathway.

### Statistical analysis

The data statistical analysis was conducted using GraphPad Prism 8 software. The *T*-test was used for comparisons between two groups, while one-way ANOVA with the Tukey test was used for comparisons between multiple groups. A *p*-value of less than 0.05 was considered statistically significant. For transcriptome analysis, statistical analysis was performed using the software’s established analysis pipeline.

## Results

### Evaluation of salt tolerance

**Figure 2:**
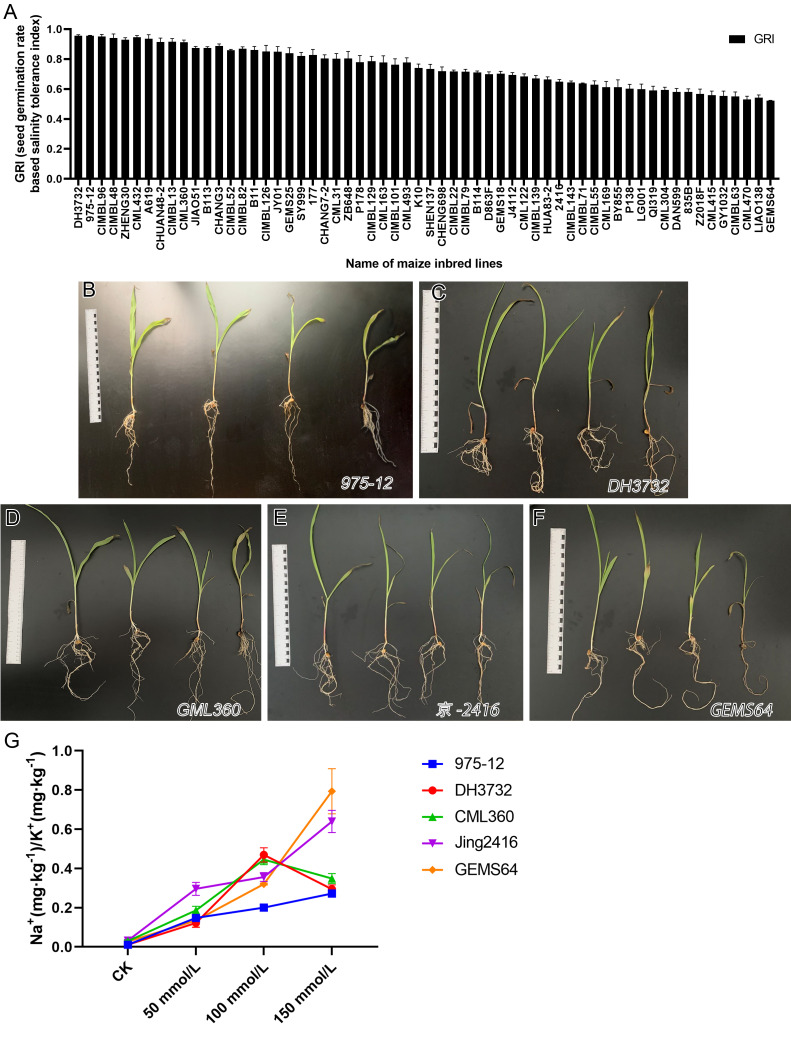
Evaluation of salt tolerance. (A) Determination of Germination Index (GRI) of 60 maize inbred lines. GRI = germination rate of salt-treated seeds/germination rate of seeds under control condition. The seeds of 60 maize lines were tested under 100 mmol/L salt stress for the preliminary germination test (three repetitions, 150 seeds were evaluated in each repetition). According to the GRI value, “975-12,” “DH3732,” “GML360,” “Jing2146” and “GEMS64” were selected as candidate plant materials. (B to F) The phenotype map of candidate maize lines under different salt stress conditions of “975-12,” “DH3732,” “GML360,” “Jing2146” and “GEMS64”, respectively. The seedlings in each image from left to right were treated with 0 mmol/L, 50 mmol/L, 100 mmol/L and 150 mmol/L NaCl concentration gradients, respectively. Three biological repeats are set for each group. (G) Na^+^/K^+^ ratio of candidate maize seedling leaves. Three biological repeats were set for each group.

The study evaluated the GRI of 60 maize inbred lines, sorting the data from high to low. Results from Fig. 2 showed that under salt stress conditions, “975-12” and “DH3732” had the highest GRI values (95.54 each), while “GEMS64” had the lowest GRI value (52.22). Table S1 provided the original GRI data. These findings suggest that “975-12” and “DH3732” displayed salt-tolerant characteristics and were less affected by salt stress during germination, while “GEMS64” demonstrated salt-sensitive characteristics and was greatly affected by salt stress.

It is not comprehensive to judge the salt resistance of maize only by GRI. To further screen salt-tolerant and salt-sensitive materials, five plant materials with the highest, high, low, and the lowest GRI value were selected according to the GRI value: “975-12,” “DH3732,” “GML360,” “Jing2146” and “GEMS64”.

In addition, we conducted a phenotype analysis of these maize seedlings at different salt concentrations. The results revealed that salt stress significantly affected the growth of all five maize inbred lines, with greater damage observed at higher salt concentrations (Figs. 2B–2F). Based on the phenotype analysis, “975-12” (Fig. 2B) exhibited the least growth inhibition under salt stress, while “GEMS64” (Fig. 2F) was the most affected.

In order to investigate the salinity tolerance mechanisms of the selected maize lines, we measured the levels of plant Na^+^ and K^+^ content and calculated the Na^+^/K^+^ ratio. The results showed that seedlings exposed to salt stress had significantly higher Na^+^/K^+^ ratios, which increased as the NaCl concentration increased for all maize materials (Fig. 2G). Under 50 mmol/L salt stress, “Jing2146” and “DH3732” had the highest and lowest Na^+^/K^+^ ratio, respectively; under 100 and 150 mmol/L salt stress conditions, “GEMS64” and “975-12” had the highest and lowest Na^+^/K^+^ ratios, respectively. Original data for these results were provided in Table S2. Based on these findings, “975-12” was considered a salt-tolerant line, while “GEMS64” was a salt-sensitive line.

### Effects of salt stress on germination characteristics of salt-tolerant and salt-sensitive lines

**Figure 3:**
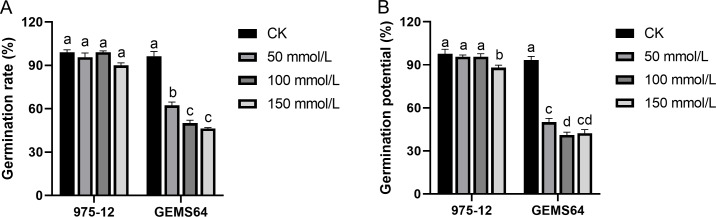
Effects of salt stress on maize germination characteristics in candidate maize materials. (A & B) The germination rate of “975-12” and “GEMS64”. Significantly different groups are indicated by diverse letters in the graphs (*p* < 0.05), whereas non-significant groups (*p* > 0.05) share similar letters. Three biological repeats were set for each group.

Under normal conditions, “975-12” and “GEMS64” had average germination rates of 99.00% and 96.33%, respectively. However, when exposed to salt stress of 50 mmol/L, their germination rates decreased to 95.67% and 62.33%, respectively. Further increasing the salt concentration to 100 mmol/L resulted in germination rates of 99.00% for “975-12” and 50.00% for “GEMS64”. At a salt concentration of 150 mmol/L, the germination rates for “975-12” and “GEMS64” were 90.00% and 46.33%, respectively (Fig. 3). All the germination original data were supplied in Table S2.

In the absence of salt stress, “975-12” and “GEMS64” exhibited comparable germination potentials of 97.67% and 93.33%, respectively. However, their germination potentials were adversely affected when subjected to salt stress. Specifically, at 50 mmol/L salt stress, “975-12” and “GEMS64” had germination potentials of 95.667% and 50.00%, respectively. At 100 mmol/L salt stress, their germination potentials were 95.667% and 41.00%, respectively. Finally, at 150 mmol/L salt stress, “975-12” and “GEMS64” had germination potentials of 88.00% and 42.33%, respectively (Fig. 3). The original data for these results can be found in Table S2 of the supplementary materials.

### Effects of salt stress on antioxidant enzymes of candidate maize inbred lines

**Figure 4:**
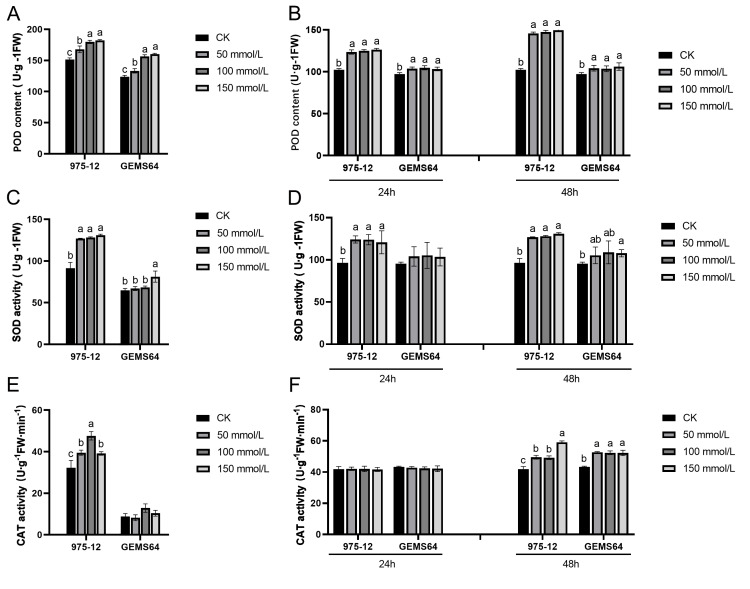
Effects of salt stress on maize antioxidant enzyme activity. (A, C and E) The POD, SOD and CAT activity at the germination period, respectively. (B, D and F) The POD, SOD and CAT activity at the seedling stage, respectively. CK, control group (0 mmol/L). Three biological repeats were set for each group. Significantly different groups are indicated by diverse letters in the graphs (*p* < 0.05), whereas non-significant groups (*p* > 0.05) share similar letters.

Under salt stress, the activity of POD in the stress group of maize was significantly higher than that in the CK group in both plumule and leaf tissues. The POD activity in plumule tissues exhibited an increasing trend with increasing salt stress concentrations (Fig. 4A). In leaf tissues, the POD activity showed a significant increase under 50 mmol/L salt stress, but there was no significant difference as the salt concentration continued to rise (Fig. 4B).

The study revealed that in the plumule tissues of “975-12”, SOD activity was significantly elevated at a salt concentration of 50mmol/L but did not exhibit significant changes with further increase in salt concentration. Conversely, in “GEMS64”, SOD activity showed a remarkable increase compared to the control group (CK) when the salt stress reached 150 mmol/L (Fig. 4C). Additionally, in leaf tissues, “975-12” demonstrated a significant change in SOD activity upon exposure to low-dose salt stress, while “GEMS64” did not exhibit any significant changes in SOD activity at different concentrations after 24 h of treatment. However, after being treated with a salt stress concentration of 150mmol/L for 48 h, “GEMS64” showed a significant increase in SOD activity compared to the CK (Fig. 4D).

In plumule tissues, “975-12” exhibited higher CAT activity than “GEMS64” at all salt stress concentrations, including non-salt stress conditions (Fig. 4E). In leaf tissues, CAT activity did not show a significant change after 24 h of salt stress in both maize inbred lines. However, after 48 h of salt stress, CAT activity in “975-12” continued to increase with increasing salt stress concentration, while in “GEMS64”, CAT activity reached its peak at 50mmol/L and did not exhibit significant changes under higher salt stress concentrations (Fig. 4F). All the POD, SOD and CAT original data were supplied in Table S2.

### Effects of salt stress on transcriptome

We conducted RNA-seq analysis of plumule tissue from “975-12” and “GEMS64” to investigate the molecular mechanisms of the salt stress response across the genome. The two inbred lines were abbreviated as “975” and “G64,” respectively, and the control and salt stress groups were labeled as “CK” and “T,” respectively. We ensured that the quality of all sequencing data met the analysis requirements (Table S3). The experimental design had a statistical power (calculated by RNASeqPower) of 69.09%, 75.36%, 75.32%, 71.48%, 69.64%, 74.73%, 71.28%, and 79.26% for 975_T *vs.* G64_T, 975_CK *vs.* G64_CK, 975_T *vs.* 975_CK, G64_T *vs.* G64_CK, 975_48 h *vs.* 975_CK, 975_24 h *vs.* 975_CK, G64_48 h *vs.* G64_CK, and G64_24 h *vs.* G64_CK, respectively (Table S4).

**Figure 5:**
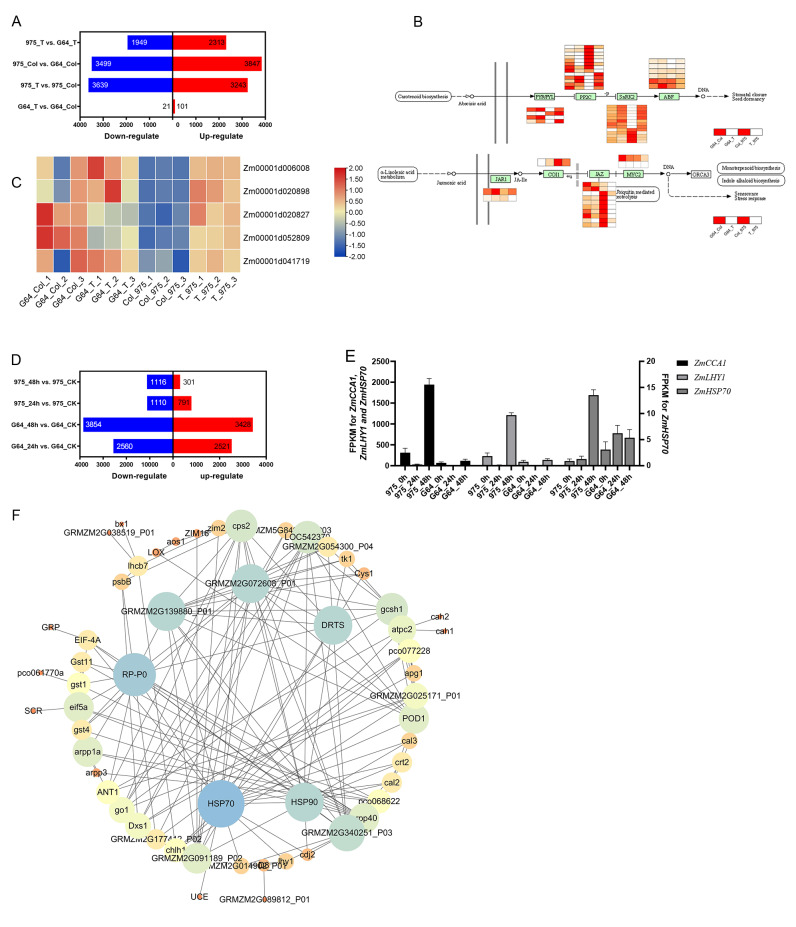
Effects of salt stress on maize transcriptome. (A) Number of differentially expressed genes (DEGs) at the germination stage. The blue and red bar indicates the number of down-regulated and up-regulated DEGs, respectively. (B) Expression of DEGs in ABA signal transduction and Jasmonic acid pathway. The redder the color is, the higher the expression level is. (C) Expression of *ZmHSP90* (candidate gene). (D) Number of differentially expressed genes (DEGs) at the seedling stage. The blue and red bar indicates the number of down-regulated and up-regulated DEGs, respectively. (E) Expression of *ZmHSP70* (candidate gene). (F) Interaction expression network of DEGs. The size of the circles indicates the number of connections in the relationship. Three biological repeats were set for each group.

In our initial analysis, we examined the RNA-seq data of plumule samples during the germination stage. By comparing G64_T *vs.* G64_CK, 975_T *vs.* 975_CK, 975_CK *vs.* G64_CK, and 975_T *vs.* G64_T, we identified 122, 6882, 7346, and 4262 DEGs, respectively (Fig. 5A). Our GO function and KEGG pathway enrichment analyses revealed that salt stress affected several metabolic pathways, such as defense response, abiotic stimulus–response, oxidation–reduction processes, photosynthesis, and plant hormone signal transduction, among others. Notably, the most enriched pathway was plant hormone signal transmission (zma04075), which includes crucial hormones and genes involved in stress response. Among the DEGs in plant hormone signal transduction, the ABA signal transduction and jasmonic acid pathways were the most affected. In ABA signal transduction pathway, *ZmPYR/PYL* and *ZmPP2C* responded positively to salt stress in “975-12” but not in “GEMS64”. More particularly in the jasmonic acid pathway, when exposed to salt stress, *ZmJAZ* did not show significant changes in “GEMS64”, but the expression level sharply decreased in “975-12” (Fig. 5B). Furthermore, we observed that the expression level of all *ZmHSP90* transcripts, which generally considered to be responsible gene to heat stress, were significantly up-regulated in “975-12” after exposure to salt stress, while they were down-regulated in “GEMS64” (Fig. 5C). Among all the *ZmHSP90* transcripts, Zm00001d020827 and Zm00001d052809 displayed contrasting expression patterns in response to salt stress across the two cultivars.

The analysis of RNA-seq data from seedling leaf samples indicated that salt stress induced changes in gene expression patterns, resulting in 5081, 7282, 1901, and 1417 differentially expressed genes (DEGs) in G64_24 h *vs.* G64_CK, G64_48 h *vs.* G64_CK, 975_24 h *vs.* 975_CK, and 975_48 h *vs.* 975_CK, respectively (Fig. 5D). Further investigating GO and KEGG function enrichment results revealed that salt stress influenced several metabolic pathways, such as those involved in abiotic stimulus–response, protein processing in the endoplasmic reticulum, and plant hormone signal transduction. Notably, under salt stress conditions, the expression levels of Zm00001eb172450 (*ZmCCA1*), Zm00001eb415770 (*ZmLHY1)*, and Zm00001eb148420 (*ZmHSP70*) showed significant differences between the two varieties (Fig. 5E). The RPKM values can be found in Table S5 of the supplementary materials.

In addition, we utilized the STRING database (https://cn.string-db.org/) to examine the interaction network of the identified genes (which were chosen based on clustering analysis and gene expression), and then created an interaction network map using Cytoscape. A total of 80 differentially expressed genes (DEGs) were included in the analysis of the interaction network. Our results revealed that ZmHSP90 and ZmHSP70 exhibit strong interactions with other genes. Furthermore, ZmPR-P0 and ZmDRTS were found to have extensive interactions (as depicted in Fig. 5F). Taken together with the transcriptome findings, our results suggest that *ZmHSPs* play a crucial role in conferring salt stress tolerance in maize, thus necessitating further investigation and verification of their functions.

### Transformation and regeneration of transgenic plants overexpressing *ZmHSP90*

**Figure 6:**
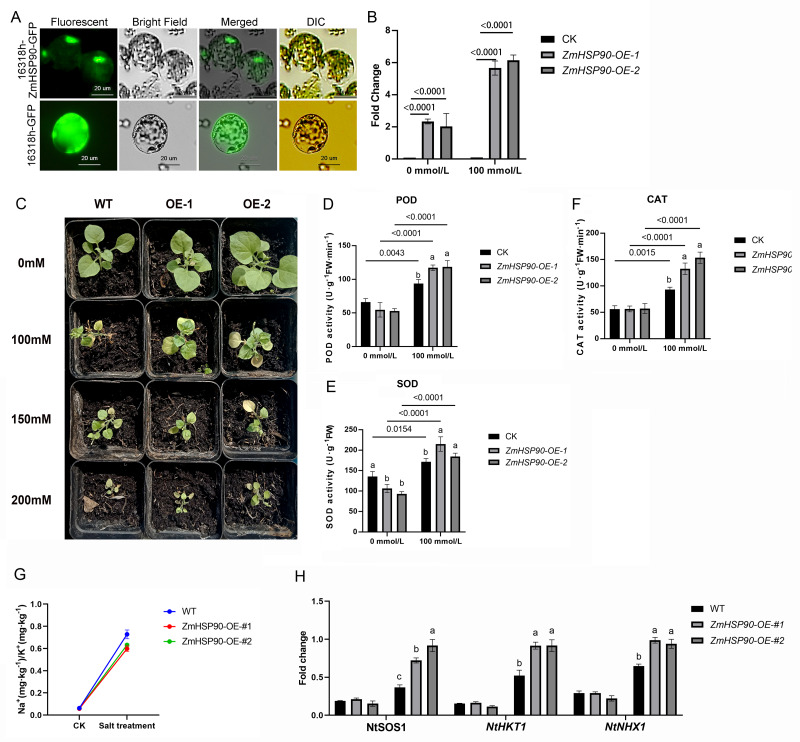
Overexpression of *ZmHSP90* in tobacco. (A) Subcellular localization of *ZmHSP90* in Arabidopsis thaliana protoplasts. Protoplasts in green fluorescence (first column) and bright field (second column) were photographed. Merged images are shown in the third column. Protoplasts with DIC are shown in the fourth column. (B) Expression of *ZmHSP90* in WT, OE-1 and OE-2. WT, wild type; OE-1, overexpression of tobacco line 1; OE-2, overexpression of tobacco line 2. (D, E and F) represents the POD, SOD and CAT activity, respectively. Values indicated by dissimilar letters are significantly different (*p* < 0.05). Three biological repeats were set for each group.

As *ZmHSP90* expression is induced by salt stress, transgenic tobacco plants were created to investigate the role of *ZmHSP90* in salt stress tolerance. Nicotiana benthamiana was selected as the model plant due to its ease of cultivation, short lifespan (∼3 months), and well-established DNA delivery methods. Additionally, tobacco has been previously used to validate maize gene functions (Fang et al., 2021; Ju et al., 2021; Sun, Wang & Xia, 2019; Wang, Wang & Xia, 2019b; Xia et al., 2018). Based on subcellular localization tests, the recombinant plasmid and empty vector were expressed in the nucleus and chloroplast, respectively. Furthermore, the labeled protein fluorescence of the 16318h-ZmHSP90-GFP recombinant plasmid (Zhang et al., 2021) was found to be exclusively detectable in the nucleus and was high, indicating that the coding protein of ZmHSP90 is a nuclear localization protein (Fig. 6A). Two transgenic lines were confirmed by PCR using primers specific to ZmHSP90, and RT-qPCR analysis showed that ZmHSP90 mRNA was only present in the transgenic plants but not in the wild type (WT) (Fig. 6B). The two lines with higher levels of *ZmHSP90* expression were then used in the stress tolerance test.

To investigate whether transgenic overexpression of *ZmHSP90* was correlated with salt stress tolerance, the WT and transgenic lines were subjected to 100 mmol/L salt stress. Lines overexpressing *ZmHSP90* demonstrated significant salt tolerance when the seedlings were grown in 100 mmol/L salt stress for 28 days (Fig. 6C). We examined the peroxidase activity of both transgenic and WT tobacco. The findings depicted that the POD increased significantly in both WT tobacco and transgenic tobacco after salt stress. The increase in POD caused by salt stress was significantly higher in *ZmHSP90* overexpression tobacco than in WT tobacco (Fig. 6D). There was no significant difference in SOD activity between the two kinds of tobacco before and after salt stress (Fig. 6E). Regarding CAT, salt treatment induced more CAT in *ZmHSP90* overexpression tobacco than in CK (Fig. 6F). Maintaining a balanced cytosolic Na^+^/K^+^ ratio has become a key salinity tolerance mechanism. We found that the balance of sodium and potassium ions was disrupted by salt stress. However, in tobacco plants overexpressing *ZmHSP90*, the adverse effects of salt stress on Na^+^/K^+^ were smaller than in WT tobacco plants (Fig. 6G). In addition, *ZmHSP90* significantly enhanced the expression levels of *NtSOS1*, *NtHKT1*, and *NtNHX1* as compared to those in the WT (Fig. 6H), suggesting that *ZmHSP90* modulates Na^+^ transporters to maintain K^+^/Na^+^ balanced in tobacco. All the transgenic related original data were supplied in Table S6.

## Discussion

During the early stages of seed development, specifically germination and early seedling growth, plants are more susceptible to the harmful effects of salinity as compared to later developmental stages (Farooq et al., 2015). When exposed to salt stress, the activity levels of most antioxidant compounds were observed to increase significantly in both the germination and early seedling stages. Antioxidant enzymes such as SOD, POD, and CAT play a crucial role in scavenging ROS in plant cells and increasing their activity is vital for enhancing plant salt tolerance (Jiang et al., 2019). However, the level of antioxidant enzyme activity was found to be closely associated with the developmental stage, the variety, and the extent of salt stress. In particular, the CAT activity in the plumule of “GEMS64” was not affected by salt stress, but it became active with an increase in the salt treatment period during the seedling stage. Meanwhile, the activity level of POD and SOD increased with an increase in the degree of salt treatment. Previous research has shown that multiple antioxidant enzymes increased in radicles and plumules in walnut under drought stress, indicating that seeds have a certain level of resistance to stress during germination. The activity of POD, SOD, and CAT demonstrated distinct regulatory patterns in the plumule. We hypothesized that the types of dominant antioxidant enzymes vary among different inbred lines under salt stress. Furthermore, we observed that salt-tolerant maize exhibits a more favorable response in terms of antioxidant enzyme activity to salt stress compared to salt-sensitive materials. This increased response of antioxidant enzymes can play a significant role in enhancing the plants’ ability to withstand salt stress and remove reactive oxygen species (ROS) from cells (Ciccarelli, Bottega & Spanò, 2019; Wang et al., 2020).

During the germination and seedling stages, we conducted further analysis on transcriptome changes in two inbred lines. Our findings indicated that several genes related to plant signal transduction and defense functions were altered before and after exposure to salt treatment. Our study found that salt stress caused significant changes in DEGs related to the Jasmonic acid (JA) and abscisic acid (ABA) pathways. When plants are exposed to environmental signals, JA is synthesized and binds to COI1 and JAZ proteins, which act as suppressors of JA-responsive transcription factors (Li et al., 2020; Shang et al., 2019). The active form of JA, JA-Ile, accumulates and promotes the binding of SCF0 to JAZ, leading to the degradation of JAZ through ubiquitination by the 26S proteasome. This results in the release of active MYC2 and initiation of gene transcription, which contributes to the plant’s response to abiotic stress (Gfeller, Liechti & Farmer, 2010; Van der Does et al., 2013). The *ZmJAZ* gene family members were generally downregulated in “975-12” but not in “GEMS64”, suggesting a potential link between the *ZmJAZ* gene family and salt tolerance in maize. Based on the data, it is possible that stable *ZmJAZ* can suppress the jasmonic acid signal continuously, while salt-induced decreases in expression may regulate the activity of the jasmonic acid signal pathway. However, further experiments are needed to confirm this hypothesis. In addition, our study found that genes in the ABA signal pathway were also closely linked to salt stress. Previous research has shown that salt stress can lead to ABA accumulation and binding to its receptor PYR/PYL/RCAR, which inactivates PP2Cs (Fidler et al., 2022). SnrK2s can then phosphorylate their target and activate them, allowing ABF/AREBs and DREB2A to enter the nucleus and bind their target DNA motif, activating the expression of target genes (Jacob, Hirt & Bendahmane, 2017). However, we observed different gene changes in the ABA pathway in different varieties under salt stress, suggesting that these genes may affect plant salt tolerance through the ABA pathway. More experiments are needed to confirm this hypothesis. Previous studies have also shown that the relationship between plant hormones and salt tolerance is complex. The findings from our study provide valuable insights into the mechanism behind plant salt tolerance and pave the way for further investigations.

In addition to these systematically reported pathways and DEGs that related to salt stress that mentioned above, we have also observed a downregulation of *ZmHSP90* expression in “GEMS64” and an upregulation in “975-12” following salt stress. HSP90 is a molecular chaperone that participates in several cellular processes, including those related to stress response (Joshi et al., 2014; Lachowiec et al., 2015). Previous studies have shown that HSP90 can be strongly induced under high-temperature, osmotic, and salt stress (Abbas et al., 2017). Our results also suggest that *ZmHSP90* plays a crucial role in the salt tolerance response of maize cultivars, particularly in salt-tolerant ones. To investigate the functions of *ZmHSP90* further, we constructed *ZmHSP90-* overexpressing transgenic tobacco. As we know, maize is a monocotyledon, and tobacco is a dicotyledon; however, tobacco has been successfully applied in the validation of maize gene functions (Fang et al., 2021; Ju et al., 2021; Sun, Wang & Xia, 2019; Wang, Wang & Xia, 2019b; Xia et al., 2018). Therefore, it is feasible to select tobacco to verify the functions of maize genes. A previous study found that HSP proteins enhanced the tolerance to abiotic stress in Arabidopsis (Mu et al., 2013), but there is little known about the roles of maize *HSP90* proteins under salt stress. Under normal conditions, *LimHSP16.45* combines with the membrane as a monomer, according to Mu et al. However, as revealed in salt-stressed transgenic Arabidopsis, the *LimHSP16.45* protein was released into the cytoplasm and bound to substrate proteins as molecular chaperones (Mu et al., 2013). This is consistent with our findings. The role of the HSP protein in response to abiotic stress is still not fully understood. Abiotic stressors in plants lead to the over accumulation of ROS, which damages proteins, lipids, carbohydrates and DNA (Gill & Tuteja, 2010). The mobilization of antioxidant enzymes has become an important mechanism for plants to scavenge ROS (Tang et al., 2019; Wang, Sun & Shi, 2019a). Could HSP90 affect antioxidant enzyme activity? According to our results, the answer is *yes*. In the current study, SOD POD and CAT activity were higher in the transgenic lines than in the WT in the presence of salt, indicating that *ZmHSP90* enhanced the tolerance to abiotic stress in transgenic tobacco due to its efficient capacity for ROS scavenging by inducing antioxidant enzymes. In Arabidopsis, the overexpression of *RcHSP17.8* enhanced SOD activity (Jiang et al., 2009), whereas the overexpression of *ZmHSP16.9* in tobacco enhanced POD, CAT and SOD activity (Sun et al., 2012). Therefore, *HSP* overexpression might enhance antioxidant enzyme activity, which may be common in plants, although more investigation is needed for verification. This may be an important way for HSP to exhibit its salt tolerance characteristics in plants. In tobacco plants overexpressing *ZmHSP90*, the adverse effects of salt stress on Na^+^/K^+^ were smaller than in WT tobacco plants, meanwhile, *ZmHSP90* enhanced the expression levels of *NtSOS1*, *NtHKT1*, and *NtNHX1* as compared to those in the salt treatment, suggesting that *ZmHSP90* participates in regulating Na^+^ transporters to maintain K^+^/Na^+^ balanced in tobacco, that might be the way that *ZmHSP90* participates in plant salt tolerance.

## Conclusions

Maize inbred lines “975-12” and “GEMS64” were salt-tolerant and salt-sensitive materials, respectively. According the transcriptome data, DEGs involved in the ABA signaling pathway and the jasmonic acid pathway exhibited significant changes, suggesting that these two pathways may play a critical role in the plant’s response to the experimental conditions and may be promising targets for further investigation. Moreover, maize *ZmHSP90* plays a role in acclimation to salt stress. The transgenic results show that *ZmHSP90* can increase the activity of antioxidant enzymes and effectively eliminate excessive ROS in tobacco plants; *ZmHSP90* enhanced the expression levels of *NtSOS1*, *NtHKT1*, and *NtNHX1* as compared to those in the salt treatment, causing the maintenance of Na^+^ and K^+^ homeostasis, suggesting that *ZmHSP90* was conducive to regulate Na^+^ transporters to maintain K^+^/Na^+^ balanced in tobacco. More works are needed to reveal the mechanism behind plant salt tolerance. These findings offer valuable insights and pave the way for further investigations into plant salt tolerance.

##  Supplemental Information

10.7717/peerj.15819/supp-1 Supplemental Information 1 Diagram of salt treatment and sampling during the seedling stage In order to avoid interference from differential genes caused by developmental differences on differential genes induced by salt stress, we chose the treatment and sampling method as shown in the diagram. The blue pots represent no salt treatment, while the pink ones represent salt treatment. Taking the flag leaves at the three-leaf stage as samples for transcriptome analysis.

10.7717/peerj.15819/supp-2 Supplemental Information 2 Effects of salt stress on Germination Rate-based salinity tolerance Index (GRI) of 60 maize inbred lines GRI = germination rate of salt-treated seeds/germination rate of seeds under control conditions. The seeds of 60 maize lines were tested under 100 mmol/L salt stress for the preliminary germination test (three repetitions, 150 seeds were evaluated in each repetition).

10.7717/peerj.15819/supp-3 Supplemental Information 3 Physical and chemical characteristics of candidate maize inbred lines Data were analyzed by Studentst-testfor comparison between two groups or two-wayANNOVAfor comparison between multiple groups (three biological repeats per group). CK, control group (0 mmol/L).

10.7717/peerj.15819/supp-4 Supplemental Information 4 Transcriptome sequencing data quality control Data were obtained by Illumina Hiseq 4000 platform. Three biological repeats per group.

10.7717/peerj.15819/supp-5 Supplemental Information 5 Sample size computation using RNASeqPower Depth, the number of reads; log2FC, log2(foldchange); FDR, false discovery rate; CV, coefficient of variation; Power, power of the test.

10.7717/peerj.15819/supp-6 Supplemental Information 6 Expression level of key DEGs Three biological repeats per group. Data were exhibited by reads per kilobase per million mapped reads (RPKM).

10.7717/peerj.15819/supp-7 Supplemental Information 7 Raw data of Fig. 6 The POD, SOD and CAT data were analyzed by Studentst-testfor comparison between two groups or two-wayANNOVAfor comparison between multiple groups. The RT-qPCR data were analyzed by 2^–^ΔΔ CT method. Three biological repeats per group.

## Additional Information and Declarations

### Data Availability

The following information was supplied regarding data availability: The data are available at GSA: CRA007406.
