# Supplementary figures and images for: Maize ZmHSP90 plays a role in acclimation to salt stress
Source: PeerJ. 2023 Oct 3;11:e15819. doi: 10.7717/peerj.15819 (PMC10557940; doi:10.7717/peerj.15819)

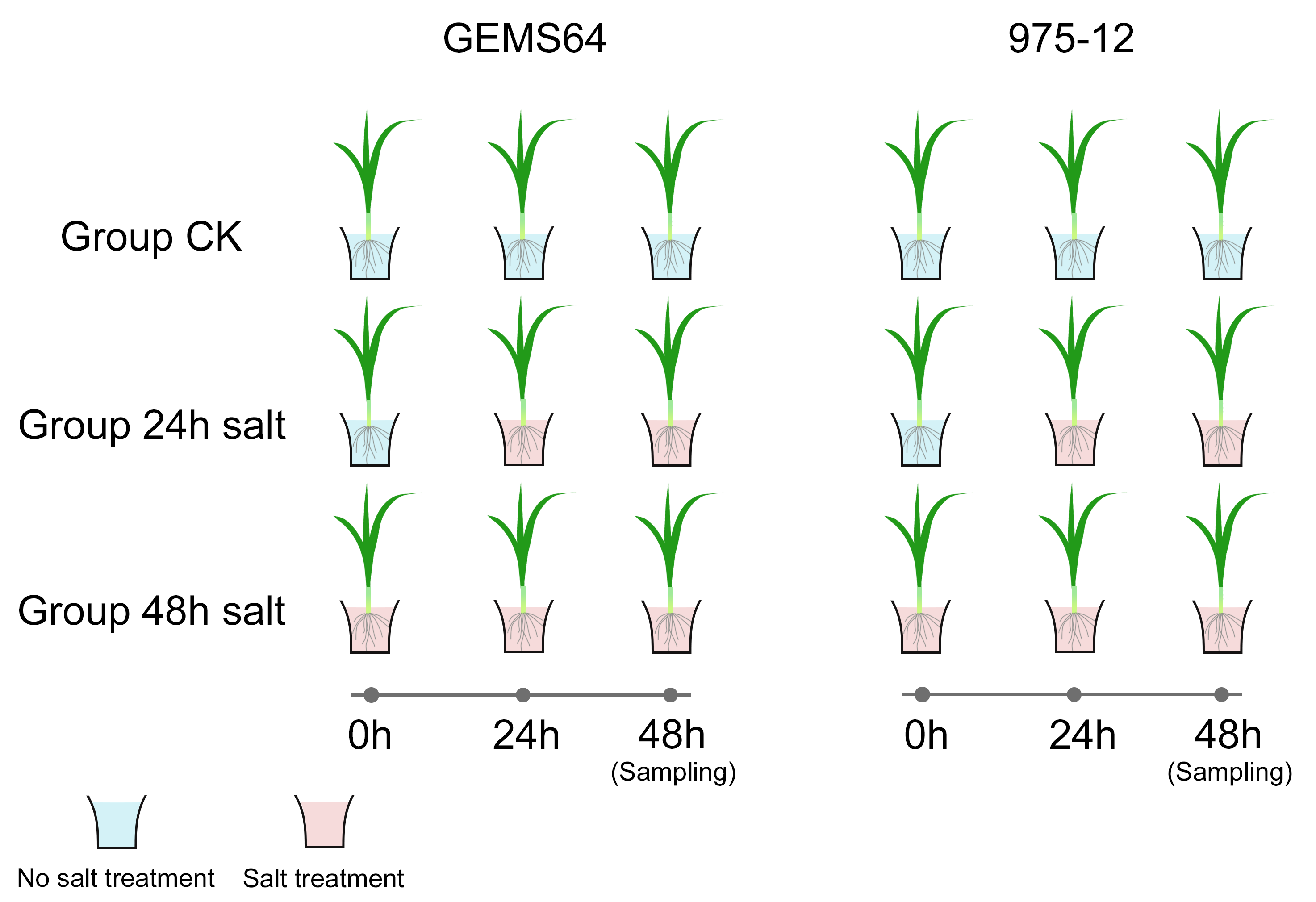

Supplement: Supplemental Information 1 — In order to avoid interference from differential genes caused by developmental differences on differential genes induced by salt stress, we chose the treatment and sampling method as shown in the diagram. The blue pots represent no salt treatment, while the pink ones represent salt treatment. Taking the flag leaves at the three-leaf stage as samples for transcriptome analysis. [file peerj-11-15819-s001.tif]
