# Supplementary material for: Maize ZmHSP90 plays a role in acclimation to salt stress
Source: PeerJ. 2023 Oct 3;11:e15819. doi: 10.7717/peerj.15819 (PMC10557940; doi:10.7717/peerj.15819)
Supplement: Supplemental Information 4 — Data were obtained by Illumina Hiseq 4000 platform. Three biological repeats per group. [file peerj-11-15819-s004.docx]

Table S3 Germ transcriptome sequencing data quality control

| Sample name | Raw reads | Raw bases | Clean reads | Clean bases | Error rate | Q20 | Q30 | GC content |
| --- | --- | --- | --- | --- | --- | --- | --- | --- |
| G64_CK_1 | 62367854 | 9.35G | 61206004 | 9.18G | 0.02% | 98.09% | 94.61% | 54.89% |
| G64_CK_2 | 67155548 | 10.07G | 65884520 | 9.88G | 0.02% | 98.04% | 94.49% | 56.87% |
| G64_CK_3 | 55975920 | 8.39G | 54545626 | 8.18G | 0.02% | 98.12% | 94.63% | 54.22% |
| G64_T_1 | 63732448 | 9.55G | 62396870 | 9.36G | 0.02% | 97.96% | 94.53% | 55.26% |
| G64_T_2 | 56901704 | 8.53G | 55633100 | 8.34G | 0.02% | 98.08% | 94.80% | 55.16% |
| G64_T_3 | 61667852 | 9.25G | 60329186 | 9.05G | 0.02% | 97.97% | 94.56% | 56.03% |
| CK_975_1 | 50311802 | 7.54G | 49075632 | 7.36G | 0.02% | 97.96% | 94.58% | 56.90% |
| CK_975_2 | 62693322 | 9.4G | 61459916 | 9.22G | 0.02% | 97.93% | 94.48% | 57.30% |
| CK_975_3 | 65887954 | 9.88G | 64100362 | 9.62G | 0.02% | 97.91% | 94.49% | 56.78% |
| T_975_1 | 68383154 | 10.25G | 67038898 | 10.06G | 0.02% | 97.94% | 94.48% | 56.04% |
| T_975_2 | 66937064 | 10.04G | 65497670 | 9.82G | 0.02% | 97.96% | 94.54% | 56.22% |
| T_975_3 | 66232458 | 9.93G | 64728548 | 9.71G | 0.02% | 97.94% | 94.47% | 56.05% |
| 975_CK_1 | 57692072 | 8.65G | 56026256 | 8.4G | 0.02% | 98.01% | 94.49% | 55.85% |
| 975_CK_2 | 73295140 | 10.99G | 71763788 | 10.76G | 0.02% | 98.13% | 94.75% | 55.63% |
| 975_CK_3 | 51985284 | 7.79G | 50946642 | 7.64G | 0.02% | 98.07% | 94.63% | 55.69% |
| G64_CK_1 | 53940972 | 8.09G | 52877522 | 7.93G | 0.03% | 97.90% | 94.18% | 53.75% |
| G64_CK_2 | 49639738 | 7.44G | 48207026 | 7.23G | 0.03% | 97.92% | 94.32% | 53.44% |
| G64_CK_3 | 55873814 | 8.38G | 54658044 | 8.2G | 0.02% | 97.96% | 94.37% | 53.63% |
| 975_24h_1 | 52737556 | 7.91G | 51508242 | 7.73G | 0.03% | 97.91% | 94.25% | 53.99% |
| 975_24h_2 | 53040772 | 7.95G | 51785382 | 7.77G | 0.02% | 98.12% | 94.73% | 53.60% |
| 975_24h_3 | 55374210 | 8.3G | 54238814 | 8.14G | 0.03% | 97.90% | 94.23% | 54.19% |
| G64_24h_1 | 52136008 | 7.82G | 50684074 | 7.6G | 0.02% | 98.15% | 94.89% | 54.61% |
| G64_24h_2 | 59593802 | 8.93G | 58199418 | 8.73G | 0.02% | 98.01% | 94.54% | 54.83% |
| G64_24h_3 | 60397172 | 9.05G | 58894544 | 8.83G | 0.02% | 98.08% | 94.69% | 54.40% |
| 975_48h_1 | 54687384 | 8.2G | 53046306 | 7.96G | 0.02% | 98.11% | 94.75% | 55.07% |
| 975_48h_2 | 62053860 | 9.3G | 60609186 | 9.09G | 0.02% | 97.96% | 94.44% | 54.67% |
| 975_48h_3 | 59578294 | 8.93G | 58025632 | 8.7G | 0.02% | 97.99% | 94.42% | 54.68% |
| G64_48h_1 | 62813362 | 9.42G | 61339224 | 9.2G | 0.02% | 98.00% | 94.51% | 55.17% |
| G64_48h_2 | 67702000 | 10.15G | 65143312 | 9.77G | 0.03% | 97.27% | 92.80% | 55.81% |
| G64_48h_3 | 58785208 | 8.81G | 57369696 | 8.61G | 0.02% | 97.94% | 94.37% | 55.70% |

Q20 Q30
